# The Effect of Trail Pheromone and Path Confinement on Learning of Complex Routes in the Ant *Lasius niger*

**DOI:** 10.1371/journal.pone.0149720

**Published:** 2016-03-09

**Authors:** Tomer J. Czaczkes, Tobias Weichselgartner, Abel Bernadou, Jürgen Heinze

**Affiliations:** Fakultät für Biologie und Vorklinische Medizin, LS Zoologie / Evolutionsbiologie, Universität Regensburg, Regensburg, Germany; University of Arizona, UNITED STATES

## Abstract

Route learning is key to the survival of many central place foragers, such as bees and many ants. For ants which lay pheromone trails, the presence of a trail may act as an important source of information about whether an error has been made. The presence of trail pheromone has been demonstrated to support route learning, and the effect of pheromones on route choice have been reported to persist even after the pheromones have been removed. This could be explained in two ways: the pheromone may constrain the ants onto the correct route, thus preventing errors and aiding learning. Alternatively, the pheromones may act as a ‘reassurance’, signalling that the learner is on the right path and that learning the path is worthwhile. Here, we disentangle pheromone presence from route confinement in order to test these hypotheses, using the ant *Lasius niger* as a model. Unexpectedly, we did not find any evidence that pheromones support route learning. Indeed, there was no evidence that ants confined to the correct route learned at all. Thus, while we cannot support the ‘reassurance’ hypothesis, we can rule out the ‘confinement’ hypothesis. Other findings, such as a reduction in pheromone deposition in the presence of trail pheromones, are remarkably consistent with previous experiments. As previously reported, ants which make errors on their outward journey upregulate pheromone deposition on their return. Surprisingly, ants which would go on to make an error down-regulate pheromone deposition on their outward journey, hinting at a capacity for ants to gauge the quality of their own memories.

## Introduction

Spatial memories are key to the survival of many animals. For central place foragers, which make repeated forays into the environment and must return to a fixed point, route memories are of particular importance. Repeated returns to the same locations provide the opportunity to form detailed memories of the goal and the route between multiple goals [1,2]. Social insects, such as bees and ants, are a convenient model in which to study navigation and homing, as they are experimentally tractable and may make frequent and repeated visits from a central nest to resource patches [2]. As such, much of what is known about route memory formation and navigation in insects has been learned from studies of social insects. Initially, social insects, such as ants, might rely on path integration to navigate back to their nest, but this is rapidly superseded by image matching mechanisms, such as alignment or positional image matching [2–4]. However, it is worth noting that path integration is not shut off completely, and insects can fall back on this information source if other information sources fail [5].

As well as possessing a well-developed route memory, many social insects can also communicate locations to their nest-mates. Honey bees famously perform the waggle dance, encoding both the direction and the distance at which a resource can be found [6]. Some ants actively lead naive workers to a food source they have found, in a behaviour termed tandem running, which may be accompanied by the deposition of a trail pheromone [7,8]. Many ants and termites, as well as some stingless bees, rely on pheromone trails in order to communicate the location of a resource [9]. However, communication of resource location is often rather inefficient. For example, in one experiment only 32% of honey bees that attended a dance successfully located the advertised food source [10]. Even in ants which rely heavily on trail pheromones, trail following may be very poor. Only 70% of Pharaohs ants (*Monomorium pharaonis*) foragers chose a branch marked by hundreds of nestmates at a bifurcation [11]. Other examples of poor trail following in ants abound [12,13], although some ants, especially those specialised in retrieving large food items, perform remarkably well [14–16]. It has been suggested that ‘errors’ may be tuned, to respond to the distribution of the resource being sought [17,18]. For example, recruitment to widely distributed resources might be followed with more error than for point-source resources. However, doubt has been cast on this hypothesis in honeybee recruitment [19]. Often, route memories are followed over pheromone trails, if route memories are available [12,20].

Social insects have developed a range of strategies for coping with navigational errors and routes which are difficult to learn. Honeybees perform learning flights when leaving the nest or a food source. The length of these learning flights decreases to zero over repeated journeys, but if a delay in finding the goal is experienced, learning flights reappear [21,22]. Learning flights are longer for more complex scenes [22]. Ants also perform such ‘turn back and look’ behaviours [3,23], and search a wider area in the face of uncertainty [24,25]. Unlike honeybees, ants walk to their food sources and many lay continuous pheromone trails on their routes, providing further options for assistance with complex navigation. For example, stepping off a pheromone trail is a good indication of a navigational error, and ants which do so reduce their speed, increase their sinuosity, perform more U-turns, and reduce pheromone deposition [26]. Ants can also respond to making errors by increasing pheromone deposition on return journeys if they have just made a navigational error and went on to correct it. This may be an attempt to provide more information for their sisters and their own future visits [27,28]. Moreover, ants which are uncertain of their route memories may reduce their pheromone deposition on outgoing journeys [27].

However, ants do not only modulate pheromone deposition in response to making errors. The presence of pheromone trails or home range markings may act as a ‘reassurance’ to ants that they are on the correct path, not only affecting their movements and pheromone deposition, but also their route learning. In a previous study, Czaczkes et al. [28] challenged *Lasius niger* ants on a doubly-bifurcating maze, and found that ants had difficulties learning alternating routes (e.g. turn left at the first bifurcation and right at the second, also later reported by Grüter et al. [29]). This in turn influences colony-level foraging decisions, by causing colonies to focus their foraging on food sources at the end of easy-to-learn routes [29]. The presence of trail pheromone reduced errors. Moreover, ants which had been trained in the presence of trail pheromone made fewer errors than ants which had been trained without pheromone, even when tested on pheromone-free mazes. Thus, the presence of trail pheromone seemed to improve route memorisation, as hypothesized by Collett and Collett [30]. However, the reason for this improvement was unclear. Collett and Collett [30] proposed two possible mechanisms which could act to improve memorisation: confinement and reassurance. The confinement hypothesis suggests that the presence of trail pheromone reduces navigational errors, and that this then translates into better route memorisation, perhaps due to no erroneous intermediate snapshot memories being made. The reassurance hypothesis suggests that the presence of trail pheromone indicates that the route is correct, and thus that memorising the current location is worthwhile.

Here, we aimed to disentangle these two possible effects, in order to see whether either, or both, mechanisms can explain the benefits of pheromone trails for route memorisation.

## Methods

### Study species

We studied eight *Lasius niger* colonies collected on the University of Regensburg campus. Colonies were housed in plastic foraging boxes (40×30×20 cm high). The bottom of each box was covered with a layer of Plaster of Paris. Each foraging box contained a circular plaster nest box (14 cm diameter, 2 cm high). The colonies were queenless with ca. 1,000 workers and small amounts of brood. Colonies were fed three times per week with Bhatkar diet, a mixture of egg, agar, honey and vitamins [31]. Colonies were deprived of food for four days prior to a trial in order to achieve uniform and high motivation for foraging. Water was provided *ad libitum*.

### Experimental design

**Fig 1:**
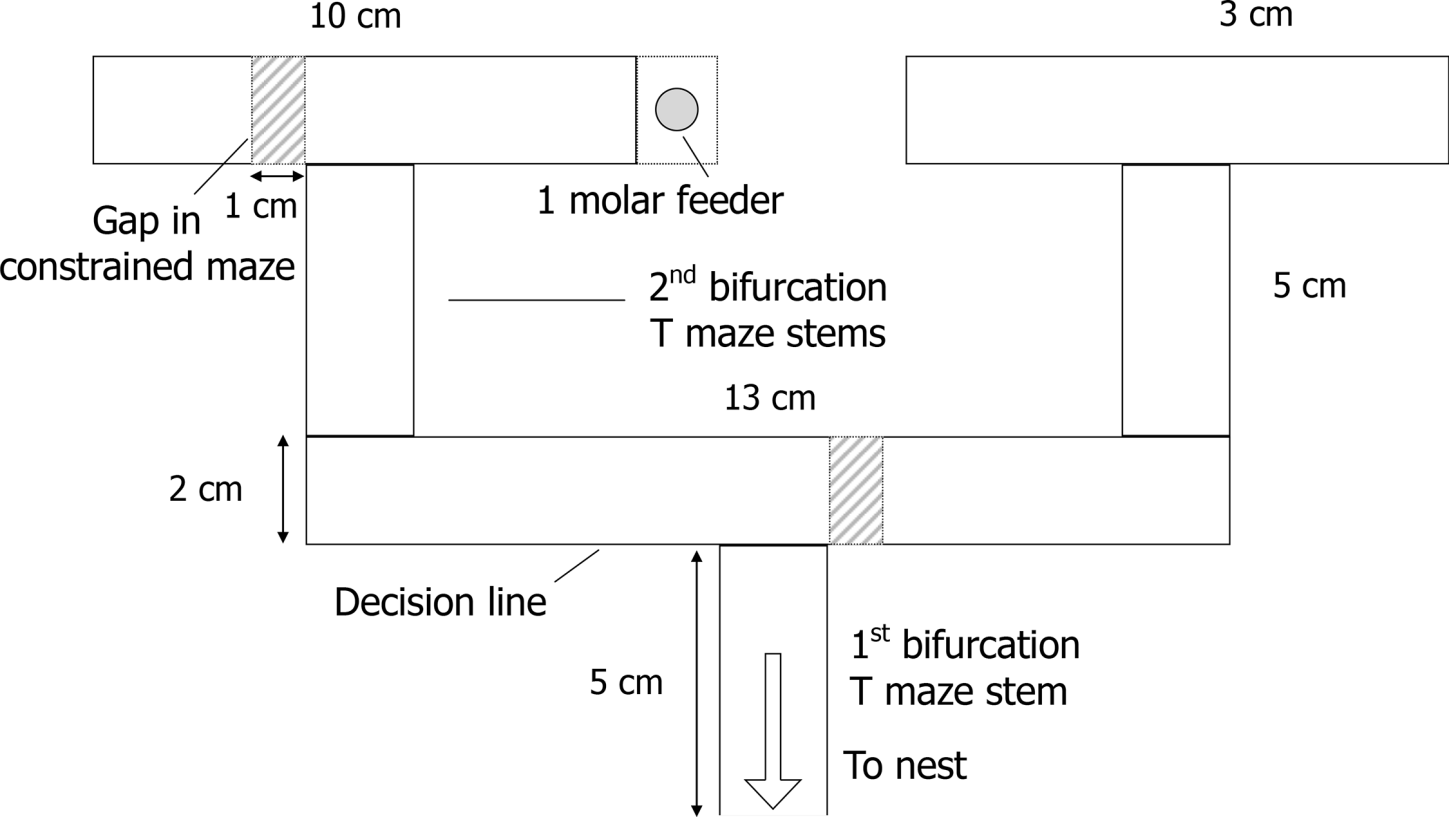
Double-bifurcation maze. Pheromone depositions were recorded on the T maze stems (shaded grey). Ants were considered to have chosen left or right at a bifurcation when they crossed the relevant decision line (dashed lines). A syrup feeder (1M sucrose) was placed on a platform at either the left-right or right-left end of the maze (left-right treatment shown). In the confined maze treatments, 1 cm gaps prevented ants from making incorrect turns (hashed sections). Adapted from Czaczkes et al. [28].

The aim of these experiments was to examine route memory formation with or without the presence of trail pheromone, and with or without constraining the foraging ants onto the correct route. *L*. *niger* rapidly learn to make a correct choice at a single bifurcation and also on repeating routes on a double-bifurcation maze (i.e. having to choose the same direction at both bifurcations) even in the absence of trail pheromones [12,28]. We therefore challenged the ants with learning an alternating route (e.g. having to turn right, then left) on a double-bifurcation maze (see Fig 1). In a previous experiment [28] *L*. *niger* ants were found to find learning such alternating routes difficult, but not impossible, even in the absence of trail pheromones. A drop of 1 molar sucrose solution was placed on a plastic platform affixed to the end of either the left-right or the right-left path. The maze used was identical to the ‘short’ maze used in Czaczkes et al. [28], in order for the results to be comparable across both studies. The various sections of the maze were covered in paper overlays, which could be replaced to remove any trail pheromone deposited. A light source was placed 2 meters to the right of the apparatus, and the experiment was carried out in an open lab space with many objects, which could act as landmarks for the ants.

Colonies were given access to the maze via a drawbridge. The first five ants to reach the feeder were individually marked with dots of acrylic paint on the abdomen, any remaining ants were removed from the apparatus, and the bridge raised to prevent further ants entering the maze. The marked ants were then allowed to make seven return from the feeder to the nest. The drawbridge was used to allow the marked ants into and out of the maze without allowing unmarked ants onto the maze. The decision at each bifurcation was recorded for every ant. Ants were considered to have made a decision if their antennae crossed a ‘decision line’ 3 cm from the centre of the bifurcation. Furthermore, the number of pheromone depositions performed by each ant on each stem section of the maze was recorded. Pheromone deposition is a very stereotyped behaviour in *L*. *niger*, and easily quantified by eye. It involves the ant pausing for ca. 0.2 seconds, backing up, and firmly pressing the tip of their abdomen onto the substrate [32].

In this experiment, any effects of trail pheromone presence directly aiding memory formation needed to be disentangled from the effect of trail pheromone constraining ants onto the correct path. To this end, four different treatments were carried out: 1) pheromone present / confined maze, 2) pheromone removed / confined maze, 3) pheromone present / open maze, and 4) pheromone removed / open maze.

To constrain ants to a path without the presence of trail pheromones, a maze identical to that in Fig 1 was used, but with 1 cm gaps preventing access to the wrong choice at the bifurcation. The use of such gaps ensured that the view the ants had from the confined maze was almost identical to the view from the open maze. Before the ants’ last visit, the confined maze was replaced with the open maze, so that the learning success of the ants could be measured.

To remove trail pheromone from the maze, the paper overlays covering it could be replaced after an ant had walked over it. To control for any disturbance caused by the removal of the overlays during this pheromone removal treatment, we sham-removed (removed and replaced) the overlays in treatments were trail pheromones were allowed. In pheromone-present treatments, the overlays were removed, and the maze cleaned with ethanol, before the ant’s last visit. This ensured that ants on the last visit had to rely on their route memory alone.

Note that on the ants’ seventh (final) visit all ants experienced the same treatment: an open maze with no trail pheromone. Each colony was tested twice on each treatment: once with the food source on the left-right end of the maze, and once with the food source on the right-left end.

### Statistical analysis

Statistical analyses were carried out in R 3.1.0 [33] using Generalised Linear Mixed Models (GLMMs) [34]. Following Forstmeier & Schielzeth (2011) we included only factors and interactions for which we had a-priori reasons for including.

As the pheromone deposition behaviour of ants heading towards the food source (outgoing) and ants returning to the nest is known to be very different [26,28,32], we analysed the behaviour of outgoing and returning ants separately. As multiple data points were collected from each individual and multiple ants were tested per colony, ant and colony identity were added as random effects, with ant identity nested inside colony identity. Binomial data (correct decision or not, pheromone deposited or not) were modelled using a binomial distribution and logit link function. Count data (number of pheromone depositions for ants which deposited pheromone at least once) were modelled using a Poisson distribution using a log link function. All P-values are corrected for multiple testing using a Benjamini-Hochberg correction [35]. The models used in the statistical analysis are provided in S1 File.

As some of the results of this study did not confirm results of a previous study [28], we carried out a simulation-based power analysis on the previous study, using the methods and R package described by Johnson et al. [36]. Specifically, we simulated a GLMM analysis based on the data of Czaczkes et al. [28] where identical experimental treatments to the current open maze / pheromone removed and open maze / pheromone remain were carried out. We only considered the first bifurcation, where a significant difference was described, and only considered the final visit of the ants [28]. The power analysis method used generates simulated datasets from generalised linear mixed-effect models, and takes random effects and distribution family into account.

### Ethical statement

This study complies with the ethical guidelines of the country where the research was carried out.

## Results

The complete statistical output and supplementary figures can be found in in S1 File. The complete dataset can be found in S1 Table.

### Decision making

**Fig 2:**
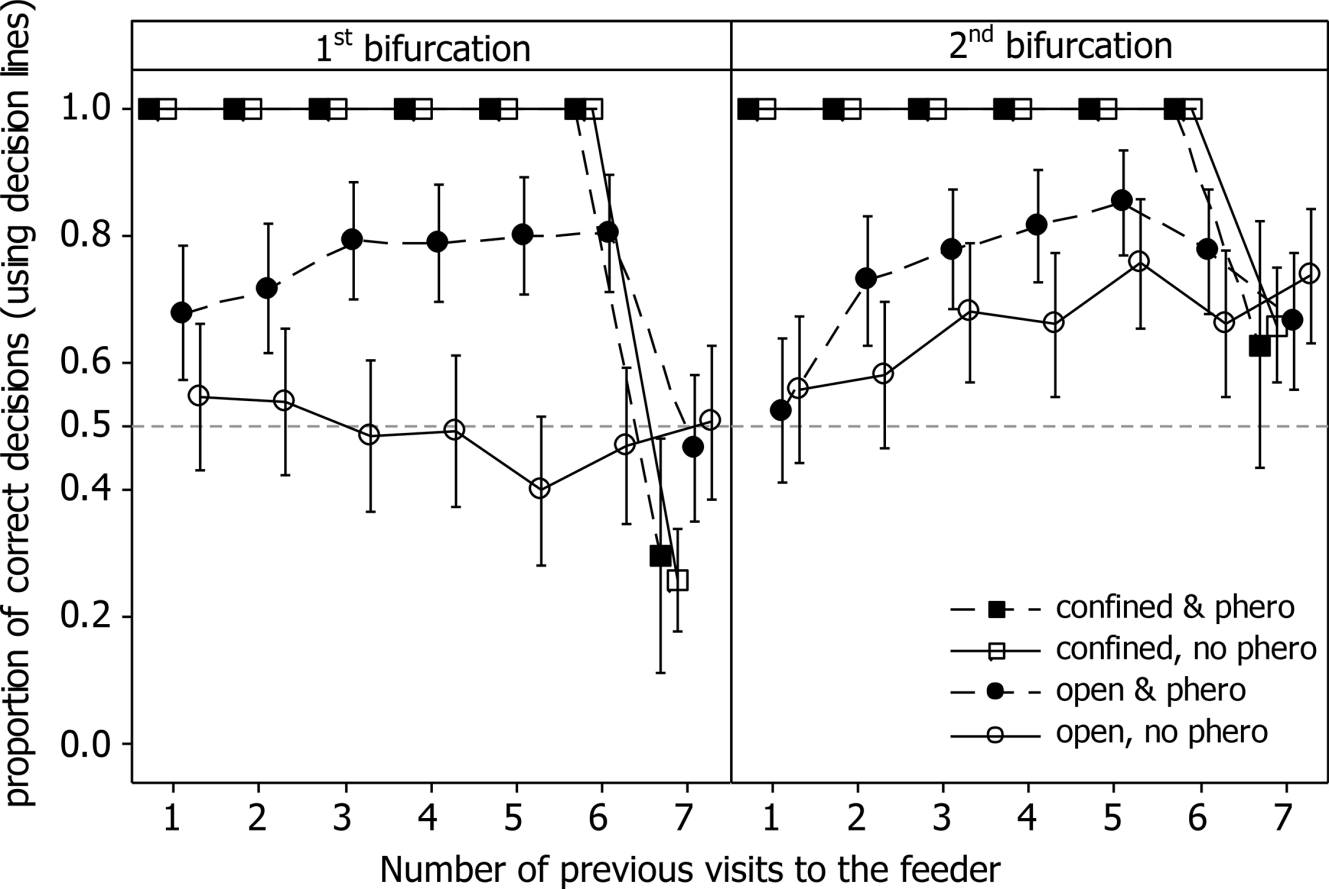
The effect of pheromone and maze treatment on learning. Ants were trained either on an open maze or on a confined maze, and pheromone was either removed or allowed to remain. Note that on the final (7^th^) visit shown, all ants are tested on an open maze with no pheromone information. The presence of pheromone during the previous training visits had no effect on learning, and confinement caused ants to make more mistakes at the first bifurcation. Symbols represent means, whiskers 95% Cis.

Contrary to expectations and in contrast to the results of Czaczkes et al. [28], neither path confinement nor the presence of pheromone, improved the route memory of ants. On the final visit to the feeder, ants which had been trained in the presence of pheromone were not more accurate than ants trained in the absence of pheromone (GLMM, Z = -0.46, P = 0.65, Fig 2). Confinement was actually detrimental to ant learning, with ants trained on a confined maze making more mistakes at the first bifurcation than ants trained on a non-confined maze (Z = -3.3, P = 0.0017, Fig 2). Even when only considering data from the ‘pheromone removed’ treatment, we find that ants in the confined treatment made more errors than ants in the open treatment (Z = 3.21, P = 00018). As in Czaczkes et al. [28], overall, ants made fewer errors on the second bifurcation (Z = 5.15, P < 0.0001). As long as pheromone was present (visits 1–6 pheromone allowed treatment, see Fig 1, especially 1^st^ bifurcation), accuracy remained high, but when pheromone was removed on visit 7 accuracy dropped to the level of the ‘pheromone removed’ treatment.

By comparing the performance of ants on the penultimate and final visit, we examined the effect of pheromone presence on decision accuracy, as there is only a small difference in experience between the two visits. Only data from the open maze treatment were compared, as ants on the confined maze treatment could make no choice on the 6^th^ visit. Accuracy dropped from the 6^th^ to the 7^th^ visit in trials where pheromone trails were allowed, but not when pheromone trails were continually removed (Z = -3.37, P = 0.0017, Fig 2). Thus, trail pheromones do provide added accuracy on top of route memory, but this effect did not persist after the pheromone was removed. This is again in contrast to the findings of Czaczkes et al. [28].

### Pheromone deposition

**Fig 3:**
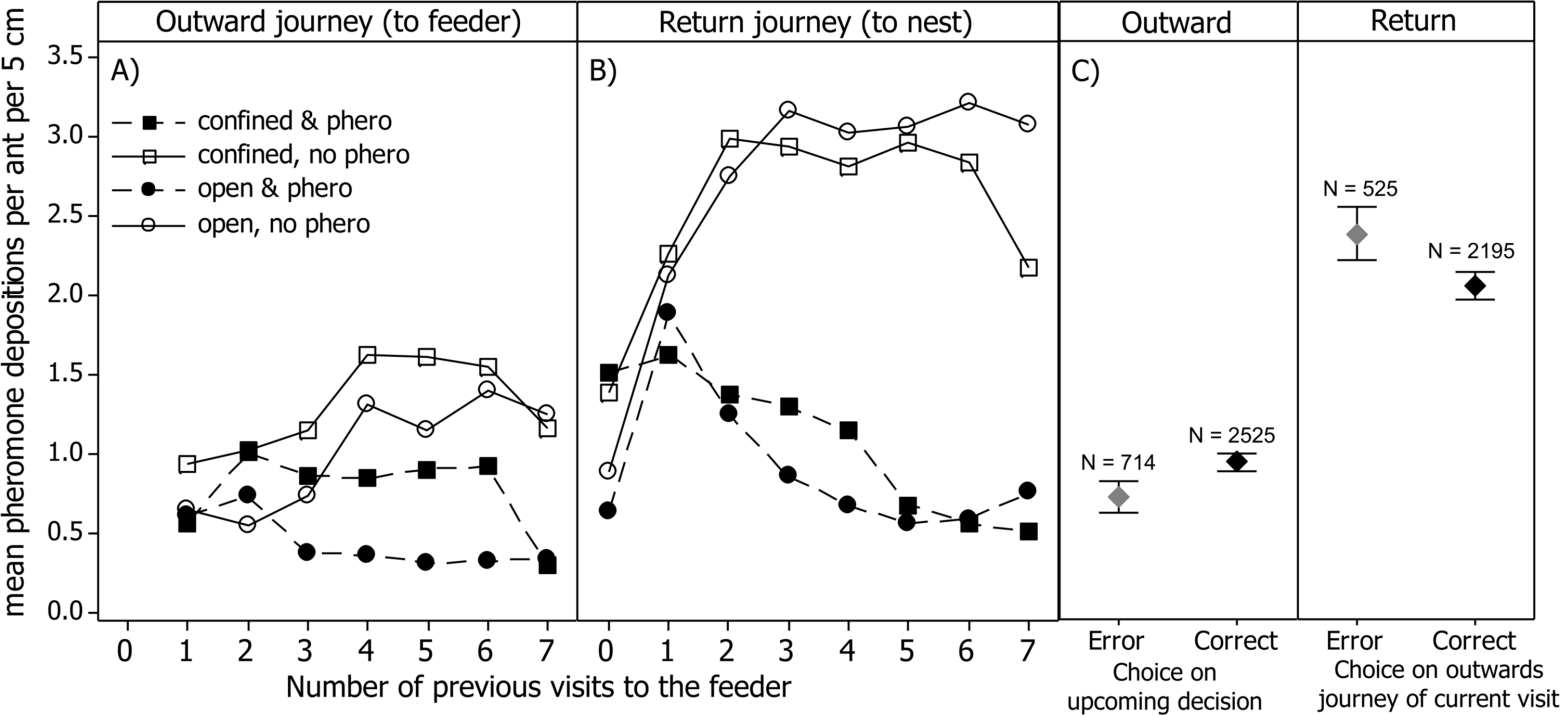
The effect of four treatments and number of previous visits on pheromone deposition (A&B), and the link between decision-making and pheromone deposition (C). Fewer pheromone depositions are performed on the outward journey in the open treatment (A). On the return journey pheromone deposition was lower if pheromone was allowed to build up, and was higher if pheromone was continually removed (B). Outgoing ants deposited less pheromone if they would go on to make a mistake on their upcoming decision (C). Returning ants deposited more pheromone if they had just made a mistake on the current visit (C). This is driven by the likelihood of depositing pheromone at all, rather than by pheromone deposition intensity (fig A in S1 File). Data from both ants which did and did not deposit pheromone are merged in this figure. Data from all open treatments, and both bifurcations, have been merged for clarity. Symbols represent means, whiskers 95% CIs. Whiskers have been omitted in A and B for clarity.

As homing and foraging are very different behaviours, and since *Lasius niger* always deposit more pheromone on their return (to nest) than their outward (to food) journeys (see Fig 3), we analysed each travel direction separately. Pheromone deposition behaviour is analysed in terms of both frequency (the proportion of ants depositing pheromone), and intensity (the number of pheromone depositions performed by depositing ants).

On the return journey to the nest, deposition frequency was higher when pheromone was continually removed, and lower if it was allowed to build up (Fig 3 & Figure A1 in S1 File, Z = 8.52, P < 0.0001). This pattern is also seen in deposition intensity; intensity is higher when pheromones are continually removed (Fig 3 & Figure B1 in S1 File, Z = 16.0, P < 0.0001). These results are similar to findings reported in Czaczkes et al. [28] and elsewhere [32].

On the outward journey (towards the food) we found no strong patterns in the data in terms of deposition frequency. However, deposition intensity was higher when pheromone was continually removed (Z = 3.54, P = 0.001, Figure B1 in S1 File). Deposition intensity was higher confined maze, and lower in the open maze (Z = 5.76, P < 0.0001, see Figure B1 in S1 File).

We also found a link between the decisions ants made, or would make, and their likelihood of depositing pheromone. As previously reported [27,28], ants which had made a navigational error on their outward journey were more likely to deposit pheromone on their return journey (Z = 3.873, P = 0.0001, see Fig 3C). Although a significant increase in deposition intensity was detected (Z = 2.02, P = 0.043), the effect size was so small that this pattern can be discounted (Figure B2 in S1 File) [37]. More surprisingly, the pheromone deposition frequency of ants travelling *towards* the food could predict their future choice: ants which would go on to make a navigational error showed lower deposition frequency than ants which went on to make a correct decision (Fig 3C outward, Z = 2.73, P = 0.0063). Deposition intensity of outgoing ants was not affect by whether they would go on to make an error or not (Z = 0.81, P = 0.42, Figure B2 in S1 File). This pattern of pheromone deposition predicting path choice accuracy has also been previously reported [27].

### Power analysis

The power of the test describing a supportive role for pheromones in memory formation in Czaczkes et al [28] was high, with an 80.3% chance of uncovering the pattern described (95% confidence interval for this estimate 78%–83%). The sample sizes in Czaczkes et al [28] were marginally lower than the samples used in the current study (58 vs 69 when pheromone removed, 63 vs 75 when pheromone remained).

## Discussion

While there were many strong similarities between the results of the current experiments and those of Czaczkes et al. [28], the result which motivated the current experiments was not replicated. Although the open maze treatments with and without pheromone duplicated exactly the methods used in Czaczkes et al. [28], we found no evidence that trail pheromone improves or confinement aids route memorisation. In Czaczkes et al. [28], performance on the first bifurcation, where most errors occur, was significantly improved by the presence of pheromone during training visits, even when pheromone was removed on the final test visit. In this study, the benefits of trail pheromones disappeared when pheromone was removed. Moreover, error rates were higher when the ants were confined during training, and indeed the choices of the ants were worse than random, with under 50% correct decisions on the first bifurcation. The reason behind this contradiction in results between the current experiment and Czaczkes et al. [28] is unclear. While the two experiments were performed in different labs, at different times, by mostly different experimenters, and with ants from different countries, we can conceive of no *a-priori* reasons for the different results. In most other respects, including the accuracy and learning trajectories of ants which were denied access to trail pheromone, the results of this study and of Czaczkes et al. [28] correspond well. The relevant tests in Czaczkes et al. [28] had a high power (80.3%), and the current tests in this study would have had a higher power still, as their samples sizes were somewhat larger. Nonetheless, it may well be that either result represents a type I or type II error, even though the odds of falsely finding no difference in the current study is small. We thus urge caution in either accepting the conclusion that pheromones support route memory formation or in rejecting it. While somewhat unsatisfactory, this highlights the value in replicating experiments.

Other results from this experiment strongly support previous findings. These include the poor learning of alternating routes, the propensity for making errors on the first bifurcation, and presence of trail pheromones reducing pheromone deposition. While the down-regulation of pheromone deposition in response to pheromone presence is a robust finding, its role is somewhat unclear. It was hypothesised that such a negative feedback effect would prevent ant colonies from becoming fixated on initially discovered food sources. However, Czaczkes [38] modelled *L*. *niger* foraging and found no effect of such a negative feedback effect on colony foraging flexibility. By contrast, a different negative feedback effect–the reduction of pheromone deposition in response to encounters with nestmates [39]–was found to have a strong colony level effect. Reduction in pheromone deposition may be performed in order to save metabolically expensive pheromone [40], or may simply be a side-effect of ants walking faster on pheromone trails [26,32].

Ants in all treatments made more errors on the first bifurcation, and indeed, there is no evidence that the ants showed any learning of the first bifurcation over the course of the 6 visits in the absence of pheromones (see Fig 2). The propensity for making errors on the first bifurcation is consistent with the ants attempting to carry out positional image-matching, [3,41,42], in which insects navigate towards a salient landmark. This would lead them in the wrong direction on the first bifurcation, assuming a single snapshot memory at the end of the trail, as to make a correct decision at the first bifurcation the ants must face away from the view faced on the final approach to the feeder. The error pattern is not consistent with the use of path integration for the ‘confined’ treatment ants, as the goal is almost straight ahead when at the first bifurcation. Thus, we would expect an error rate of ca. 50%, not the observed ca. 70%. More recent models of ant navigation, which assume continuous view acquisition instead of periodic snapshots, also do not seem to predict this error pattern [43–45]. However, conclusions based on positional image-matching should be made with caution, as most such experiments are carried out on the scale of meters, while the current experiment involves much shorter distances. This may mean not enough visual differences accrue between the bifurcation points, although *L*. *niger* regularly forage in environments (such as plants) with complexity on this scale. An alternative explanation for this pattern of data is that the ants could see the sugar feeder and simply travelled towards it. From the first bifurcation the feeder is almost directly ahead, which would result in a close to 50/50 choice of branch. If the wrong branch is chosen, this quickly becomes apparent, and the ants can correct their path. From the second bifurcation the feeder direction is unambiguous, resulting in higher accuracy. It is unlikely the ants could smell the sucrose solution, as sucrose is non-volatile, and so the vapour emanating from a water/sucrose solution will only consist of water vapour.

Contrary to expectations, preventing ants from making errors was in fact detrimental to the accuracy of their choices, resulting in many more errors being made on the first bifurcation. The ability of *L*. *niger* ants to accurately return to the nest has also been reported to improve if ants initially experienced a dead-end on an alternate route [46]. Our results therefore shed doubt on the confinement hypothesis, even if the reassurance hypothesis cannot be confirmed. More interestingly perhaps, is why preventing ants making errors should result in poorer decision accuracy. The confinement treatment prevented ants from making errors followed by U-turns in which they return to the junction facing the correct view. It could be that it is at this point that learning of the ‘counter-intuitive’ first bifurcation occurs, and that by preventing this, we prevented learning. Indeed, there is no evidence than any learning took place in the confined maze. Alternatively, being unable to make errors may have allowed the ants to learn a simpler rule–keep walking forward as much as possible. Such an effect has been reported in the Australian ant *Melophorus bagoti* when it is trained to a feeder under confined conditions [47]. Alternatively, ants on a confined maze which are suddenly given the opportunity to explore the open maze may prioritize exploration over foraging, and thus deliberately head away from their previous route. This, however, seems unlikely, as the colonies were very hungry, and the ants had just experienced six short and rewarding visits to a high quality food source via the previous route.

If errors are necessary for learning, this would cast much of the apparent ‘errors’ reported in ant navigation studies–especially during pheromone trail following, but also when following memories–in a new light [48–50]. Some errors may be deliberately made in order to more effectively memorise the route. The concept of ‘tuned errors’ has been raised, and rejected, in the honey bee waggle dance [18,19], but that does not preclude this explanation for errors in other systems, such as ant foraging [17].

Ants sense, and respond to, navigational errors in a variety of ways. The ants in this study responded to making navigational errors by modulating the amount of pheromone they deposited. When returning to the nest, ants which had made a navigational error on their outward journey deposited more pheromone *on their return journey* than ants which had made a correct decision. Such a pattern was also described by Czaczkes et al. [28], and more recently by Czaczkes and Heinze [27]. This is reasonable, as by doing so ants on difficult to learn routes will provide more information for their sisters and their own future visits. More surprisingly, ants on their *outward journey*, which would *go on to* make an error, were less likely to deposit pheromone than those that would *go on* to make a correct decision. Again, similar findings were found in a different experimental set up [27]. In line with these results, the intensity of outgoing pheromone deposition was lower in the ‘open’ maze treatments, where mistakes are possible. As in Czaczkes & Heinze [27], we cautiously attribute these patterns to a metamemory-like ability. In other words, the ants seem to be able to gauge the accuracy of their own memories, and respond appropriately, depositing more pheromone the more confident they are in their memories. Metacognition is usually considered to require extremely developed cognitive abilities, and has previously only been reported in vertebrates such as apes, dolphins, and rats [51–54]. However, the study of metacognition is fraught with difficulties, and incontrovertible proof of such a cognitively demanding ability is difficult to achieve. In this study, causation cannot be ensured, as it is for example possible that a subset of ants are both more likely to make errors and less likely to deposit pheromone (although this is to some extent controlled for by adding ant identity into the statistical analysis). Many studies, including a study on honey bee metacognition, are forced to stop short of claiming an unambiguous demonstration of metacognition due to limitations in the experimental design [55,56]. While our experimental design avoids many common pitfalls, it was not intended to unambiguously demonstrate metamemory capabilities in ants. Alternate, mechanistic explanations may be found to explain the data presented. We refer to this ability as metamemory-like, as we do not believe active self-reflection is necessary to produce the patterns observed. These patterns could arise if ants on their outward journey deposit pheromone proportionally to the strength of their memory trace, or how well the image they see matches their remembered view on the way to the food source. We thus do not attempt to claim incontrovertible proof of metamemory, although the parallel findings of similar abilities in honeybees, and near-identical results being repeated in two different experiments with ants, are very suggestive.

In this experiment, we revisited a previous experimental design in order to gain further insights into a previously described phenomenon. This was a productive course of action: on the one hand, we uncovered ambiguity in the results, calling previous findings into question–am important endeavour in science [57]. On the other hand, we supported other findings, allowing high level of confidence in these results. We also uncovered unexpected patterns in the findings, pointing towards a metamemory-like ability. Lastly, our data suggests that making errors is important when learning complex routes, and that when it comes to route learning, making errors is a key part of successful learning.

## Supporting Information

S1 File Supplementary figures, and full statistical output. (PDF)

S1 Table Complete raw data. (XLSX)
