# Supplementary material for: The Effect of Trail Pheromone and Path Confinement on Learning of Complex Routes in the Ant Lasius niger
Source: PLoS One. 2016 Mar 9;11(3):e0149720. doi: 10.1371/journal.pone.0149720 (PMC4784821; doi:10.1371/journal.pone.0149720)
Supplement: S1 File — (PDF) [file pone.0149720.s001.pdf]

Online supplement for Czaczkes, Weichselgartner, Bernadou and Heinze:

The effect of trail pheromone and path confinement on learning of complex routes in the ant *Lasius niger*

Contents

Supplementary figures 2
Supplementary statistical details 3
Decision making data – final (7th) visit 3
Decision making data – comparing the penultimate (6th) and final (7th) visits 3
Pheromone deposition data 3
Deposition intensity (number of depositions) 4
The effect of path decision making on pheromone deposition 4
Deposition probability 4
Deposition intensity 5

Supplementary figures

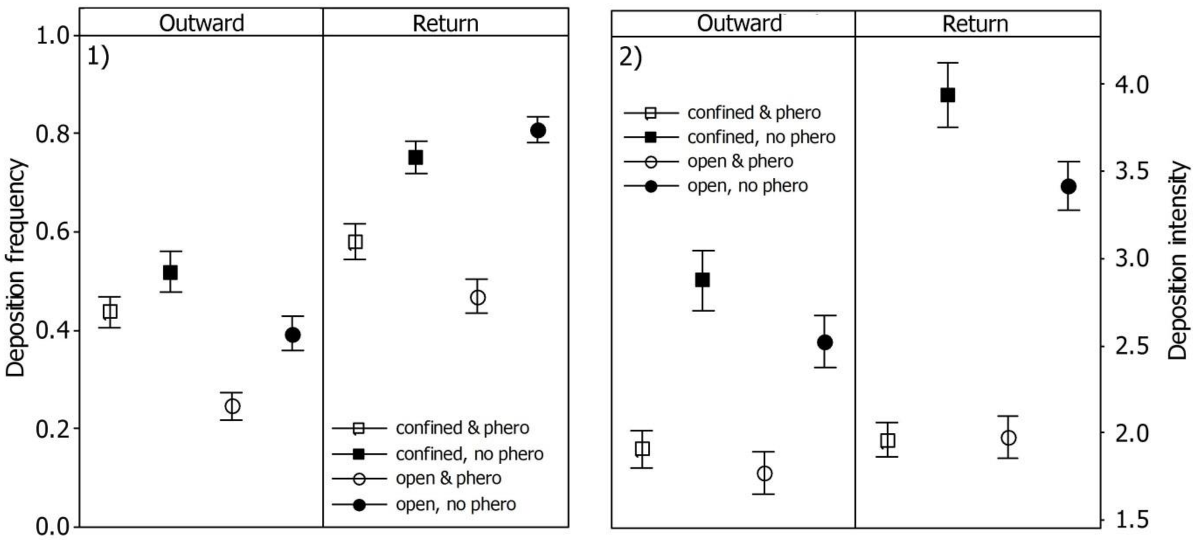

Figure A - The effect of pheromone and maze treatment on pheromone deposition. 1) deposition frequency (the proportion of ants depositing pheromone). 2) Mean deposition intensity (the number of depositions by ants making at least one deposition). Data for all visits as shown, except for the first outward visit (where ants do not know that food is available, and so do not deposit pheromone at all). Symbols are means, whiskers are 95% CIs.

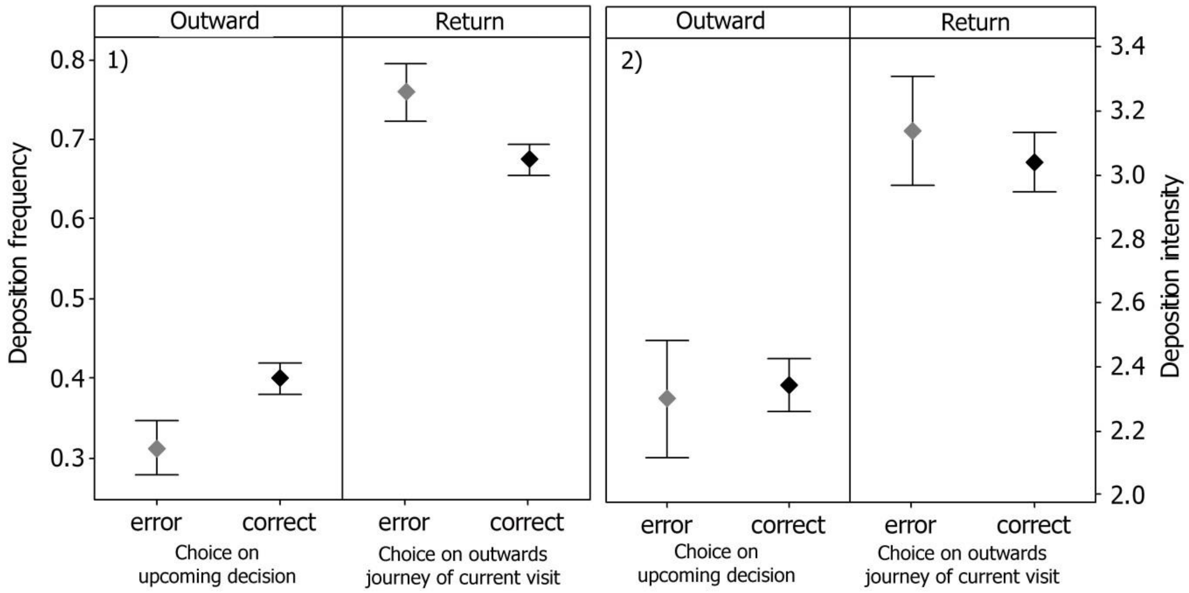

Figure B - Making errors affects pheromone deposition. 1) Deposition frequency. 2) Deposition intensity. Symbols are means, whiskers are 95% CIs.

Supplementary statistical details

Decision making data – final (7th) visit

As the decision data is binomial, a binomial distribution family (with logit link function) is used. The main model formula used was:

```
Decision on final visit = maze treatment * bifurcation +
         pheromone treatment * bifurcation +
        (random effects: ant nested within colony)
```

This provided the following model fit:

```
Fit: glmer(formula = correct ~ mazetreatment * bifurcation + pherotreatment *
    bifurcation + (1 | Colony/ant), data = onlytrip7, family = binomial)

Linear Hypotheses:
                                           Estimate Std. Error z value Pr(>|z|)
(Intercept) == 0                            -0.8493     0.2314  -3.670 0.000728 ***
mazetreatmentopen == 0                       0.8487     0.2548   3.330 0.001735 **
bifurcation2nd == 0                          1.7318     0.3363   5.149 1.57e-06 ***
pherotreatmentremoved == 0                  -0.1158     0.2512  -0.461 0.644801
mazetreatmentopen:bifurcation2nd == 0       -1.0298     0.3697  -2.786 0.008010 **
bifurcation2nd:pherotreatmentremoved == 0    0.4537     0.3665   1.238 0.258913
```

Decision making data – comparing the penultimate (6th) and final (7th) visits

This comparison was made to explore the role of trail pheromone in navigation. Only data from the open maze treatment was used, as ants could not make a decision in the closed maze treatments. The model formula used was:

```
Decision ~ pheromone treatment * trip (6th or 7th) * bifurcation
         + (random effects: ant nested within Colony)
```

This provided the following model fit:

```
Linear Hypotheses:
                                                   Estimate Std. Error z value Pr(>|z|)
(Intercept) == 0                                     1.4305     0.3043   4.700 2.08e-05 ***
pherotreatmentremoved == 0                          -1.5448     0.3895  -3.966 0.000195 ***
Ftrip7 == 0                                         -1.5640     0.3805  -4.110 0.000158 ***
bifurcation2nd == 0                                 -0.1692     0.4119  -0.411 0.681188
pherotreatmentremoved:Ftrip7 == 0                    1.7118     0.5129   3.337 0.001692 **
pherotreatmentremoved:bifurcation2nd == 0            0.9626     0.5440   1.770 0.102390
Ftrip7:bifurcation2nd == 0                           1.0004     0.5336   1.875 0.097299
pherotreatmentremoved:Ftrip7:bifurcation2nd == 0    -0.7760     0.7373  -1.053 0.334356
```

Pheromone deposition data

As pheromone deposition is much higher when ants are returning from a food source than when outgoing from the nest, pheromone deposition was investigated separately according to travel direction. To disentangle the choice of depositing pheromone at all from the modulation of pheromone deposition, we consider both whether ants deposit pheromone or not (binomial data), and then the number of depositions performed by ants depositing pheromone (count data). Count data was modelled using a Poisson distribution.

In all cases we used a similar model formula:

```
Pheromone deposited? OR # pheromone depositions =
  pheromone treatment + maze treatment + trip +
                    bifurcation
  + (random effects: ant nested within colony
```

This provided the following model fits:

Ants returning to the nest:

Deposition frequency (probability of depositing pheromone at least once)

	Estimate	Std. Error	z value	Pr(>|z|)	
(Intercept) == 0	0.086821	0.231409	0.375	0.839	
pherotreatmentremoved == 0	1.376169	0.161472	8.523	<2e-16	***
mazetreatmentopen == 0	-0.152054	0.160906	-0.945	0.574	
trip == 0	-0.004601	0.022654	-0.203	0.839	
bifurcation2nd == 0	0.120090	0.087755	1.368	0.428	

Deposition intensity (number of depositions)

	Estimate	Std. Error	z value	Pr(>|z|)	
(Intercept) == 0	0.676134	0.055488	12.185	< 2e-16	***
pherotreatmentremoved == 0	0.613561	0.038454	15.956	< 2e-16	***
mazetreatmentopen == 0	-0.069271	0.037121	-1.866	0.062	.
trip == 0	-0.018283	0.007209	-2.536	0.014	*
bifurcation2nd == 0	0.128944	0.026963	4.782	2.89e-06	***

Ants outgoing towards the food:

Probability of deposition

Linear Hypotheses:

	Estimate	Std. Error	z value	Pr(>|z|)	
(Intercept) == 0	-0.286272	0.172670	-1.658	0.162	
pherotreatmentremoved == 0	0.545662	0.154180	3.539	0.001	**
mazetreatmentopen == 0	-0.902272	0.156723	-5.757	4.28e-08	***
trip == 0	-0.021644	0.020026	-1.081	0.350	
bifurcation2nd == 0	-0.002639	0.079266	-0.033	0.973	

Deposition intensity (number of depositions)

	Estimate	Std. Error	z value	Pr(>|z|)	
(Intercept) == 0	0.693330	0.056598	12.250	<2e-16	***
pherotreatmentremoved == 0	0.380705	0.043443	8.763	<2e-16	***
mazetreatmentopen == 0	-0.113261	0.043070	-2.630	0.0142	*
trip == 0	-0.001507	0.010067	-0.150	0.8810	
bifurcation2nd == 0	-0.085112	0.037873	-2.247	0.0308	*

The effect of path decision making on pheromone deposition

Here, we explored how having just made an error, or being about to make an error, affects pheromone deposition. First, we examine the probability of ants to deposit any pheromone at all. We then examine pheromone deposition intensity by considering the number of depositions performed by ants depositing at least one dot of pheromone. Again, we consider journey direction (return or outwards) separately.

Deposition probability

As the response is binomial (deposited or did not deposit) a binomial distribution family is used. The model formula was:

```
Pheromone deposited? = decision (correct/incorrect)
    + (random effects: ant nested within colony
```

The model fits were:

Return journey (to nest)

	Estimate	Std. Error	z value	Pr(>|z|)
(Intercept) == 0	1.7802	0.2481	7.176	1.44e-12
correct.dec.line == 0	-0.6471	0.1671	-3.873	0.000107

Outward journey (to food)

```
                       Estimate Std. Error z value Pr(>|z|)
(Intercept) == 0        -0.7082     0.1526  -4.642 6.89e-06
correct.dec.line == 0    0.3216     0.1176   2.734  0.00626
```

Deposition intensity

As this is count data, a poisson distribution family was used. The model formula was:

```
# pheromone depositions = decision (correct/incorrect)
      + (random effects: ant nested within colony
```

The model fits were:

Return journey (to nest)

```
                       Estimate Std. Error z value Pr(>|z|)
(Intercept) == 0         0.7067     0.1270   5.566 5.22e-08
correct.dec.line == 0   -0.2054     0.1016  -2.022   0.0432
```

To food

```
                       Estimate Std. Error z value Pr(>|z|)
(Intercept) == 0        -0.2495     0.1644  -1.517    0.258
correct.dec.line == 0   -0.1126     0.1392  -0.809    0.419
```
